# Comparative analysis of inflammatory response on post resuscitation syndrome in extracorporeal cardiopulmonary resuscitation

**DOI:** 10.1016/j.resplu.2025.100995

**Published:** 2025-05-28

**Authors:** Kostiantyn Kozakov, Zdenek Provaznik, Maik Foltan, Jing Li, Walter Petermichl, Christian Stadlbauer, Sigrid Wiesner, Dzmitry Turtsevich, Andreas Keyser, Leopold Rupprecht, Christof Schmid, Simon Schopka

**Affiliations:** a Department of Cardiothoracic Surgery, University Medical Center Regensburg, Franz-Josef-Strauß-Allee 11, Regensburg 93053, Germany; b Department of Anaesthesiology, University Medical Center Regensburg, Franz-Josef-Strauß-Allee 11, Regensburg 93053, Germany

**Keywords:** Extracorporeal life support, OHCA, IHCA, Interleukins, Cytokins, Post-resuscitation syndrome

## Abstract

**Background:**

Extracorporeal life support (ECLS) is increasingly employed for severe respiratory or cardiac failure, alongside a rising adoption of extracorporeal cardiopulmonary resuscitation (eCPR). Despite increased adoption, limited evidence underscores possible cytokines’ pivotal role in the inflammatory response during ECLS.

**Methods:**

The study involved 546 eCPR patients using veno-arterial extracorporeal membrane oxygenation from 2013 to 2023. Categorized into in-hospital eCPR (IHCA, 358 patients) and out-of-hospital eCPR (OHCA, 188 patients) groups, a retrospective analysis explored associations among interleukin 6 (IL6), interleukin 8 (IL8), tumor necrosis factor-alpha (TNF-alpha), soluble interleukin-2 receptor (sIL2R), and pivotal post-resuscitation syndrome (PRS) components. Cytokine dynamics were assessed before and after ECLS initiation.

**Results:**

CPR-to-ECLS time was significantly longer in the OHCA group (*p* = 0.009), correlating with elevated IL6 and IL8 levels. The IHCA group showed more favorable neurological outcomes (*p* < 0.001), but presented with a higher incidence of multiple organ failure (*p* < 0.001) compared to the OHCA group. The IHCA group exhibited pronounced IL6 and IL8 levels prior to ECLS initiation significantly decreasing post-ECLS initiation (*p* = 0.01 and *p* = 0.008), whereas OHCA patients showed peak levels of IL6 and IL8 during the course of ECLS (*p* < 0.001).

**Conclusion:**

IL6 and IL8 are associated with key elements of the post-resuscitation syndrome (neurological outcome, organ dysfunction and hemodynamic status) in patients undergoing eCPR for refractory cardiac arrest, possibly providing a predictive ability of organ dysfunction in OHCA patients without a preceding pro-inflammatory burden.

## Graphical abstract

**Figure:**
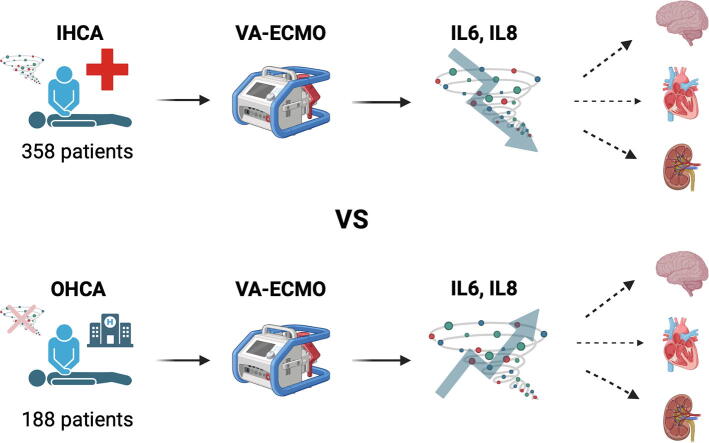

## Introduction

Extracorporeal Life Support (ECLS) has emerged as a crucial therapy for patients with severe and refractory respiratory or cardiac failure. The number of ECLS procedures remains rising, still only half of adult patients are discharged alive from the hospital.^1^ Despite the ongoing discussions about the usefulness of ECLS in an out-of-hospital setting as extracorporeal cardiopulmonary resuscitation,^2^, ^3^ the number of out-of-hospital eCPR procedures continues to grow.^4^, ^5^ In this setting, approximately one-third of treated patients is discharged from the hospital. Concerning the current resuscitation guidelines for patients with refractory cardiac arrest eCPR is recommended grade 2a, level B,^6^ pointing to the ongoing importance of the evidence base.

There is an increasing scientifical interest in comparing patients who underwent ECLS for refractory cardiac arrest within a hospital (in-hospital, IHCA) versus those who underwent the procedure outside the hospital (out-of-hospital, OHCA).^3^, ^7^, ^8^, ^9^ However, to the best of our knowledge, there have been no studies of this size that have compared the impact of cytokines and inflammatory status of patients in these two groups, as this may have an impact on the subsequent therapy strategy.

Cytokines, including interleukin 6 (IL6), interleukin 8 (IL8), soluble receptor of interleukin 2 (sIL2R), and tumor necrosis factor alpha (TNF-alpha), are key mediators of the inflammatory response.^10^, ^11^ IL6 and TNF- alpha are pleiotropic cytokines critically involved in the systemic inflammatory response following cardiac arrest, with IL-6 reflecting the magnitude of tissue injury and TNF-α may contributing to hemodynamic instability. IL-8 promotes neutrophil recruitment and endothelial activation, potentially exacerbating microvascular dysfunction. sIL-2R indicates T-cell activation and may reflect prolonged immune stimulation.

A role of these mediators in post resuscitation syndrome (PRS)^12^ and multi organ dysfunction syndrome (MODS) during ECLS^13^ has already been established. Generally elevated levels of these interleukins have been associated with a dysregulated immune response, organ dysfunction,^13^ and with a negative impact on outcomes of critically ill patients.^14^ The importance and prognostic value of the inflammatory response in OHCA patients without ECLS has also been extensively evaluated, and IL-6 in particular has been found to be associated with the severity of organ dysfunction and mortality.^15^

In order to gain insights in pathophysiology, prognostic indicators and respective differences of patients undergoing IHCA and OHCA, we analyzed the inflammatory status and it‘s impact on key elements of PRS, comparing IHCA vs OHCA patients.

## Material and methods

### Study design and definition of organ failure

A retrospective, single-center, observational study was initiated to analyze clinical aspects of the relationship between the serum cytokine levels and elements of PRS (neurological outcome, organ dysfunction and hemodynamic status) or also known as post-cardiac arrest syndrome (PCAS) as well as on survival between two groups: patients who underwent ECLS in the hospital, and patients who underwent resuscitation outside the hospital.

As all patients underwent ECLS implantation for veno-arterial extracorporeal membrane oxygenation (ECMO), all patients suffered at least one cardiocirculatory failure as a result of cardiac arrest. Detailed estimation criteria of organ failure were outlined in our previous study.^13^ Patients with dysfunction of three or more systems were defined as suffering from MODS.

The neurological status was assessed according to the (cerebral performance category) CPC score. The CPC is a 5-point scale ranging from 1 (good cerebral performance) to 5 (brain death)^16^: CPC 1 – good cerebral performance, CPC 2 – moderate cerebral disability, CPC 3 – severe cerebral disability, CPC 4 – coma or vegetative state, CPC 5 – brain death. A favourable outcome was defined as a CPC score of 1 or 2, whereas an unfavourable outcome was defined as a CPC score of 3, 4 or 5. The final assessment of the neurological status was carried out at discharge from the hospital or, in the case of suspected cerebral death, following radiological examination and confirmation of brain death.

For cumulative assessment of inotropic and vasopressor support, the vasoactive-inotropic score (VIS)^17^ was estimated.

### Indication and management of ECLS

Initiation of ECLS was carried out in patients undergoing cardio-pulmonary resuscitation (CPR) according to the guidelines of the European Council of Resuscitation, which was performed for at least 10 min without return of spontaneous circulation or sufficient heart rhythm.

The eligibility criteria for implementing extracorporeal cardiopulmonary resuscitation (eCPR) in an out-of-hospital setting were as follows: the cardiac arrest (CA) event was witnessed, basic life support (BLS) has already been initiated by non-medical personnel, and advanced cardiac life support (ACLS) was performed in accordance with the guidelines set by the European Resuscitation Council. eCPR was not initiated in cases where the patient had a known terminal malignancy, in cases of traumatic injury accompanied by uncontrolled bleeding, in cases of unwitnessed CA, or if there was a valid and credible statement from the patient expressing their refusal to receive life-prolonging treatments. The age of the patient was not considered as contraindication.

In all patients in the OHCA group, ECLS was initiated outside the hospital, i.e. in the pre-hospital setting. ECLS team activation was initiated via the Public Safety Answering Point at the onset of resuscitation. After on-site assessment, the decision for ECLS initiation was made, which we believe improves logistics and shortens the time from CPR to ECLS. In contrast, ECLS implantation in the IHCA group was performed in the hospital (e.g. intensive care unit, cardiac catheterisation laboratory, emergency department, etc.).

Arterial cannulation was performed with cannulas inserted into the femoral artery. The venous drainage was accomplished by cannulas through the femoral vein.

Detailed anticoagulation, flow, catecholamines and fluid management as well as cytokines analysis were described in our previous study.^13^

### Analysis of cytokines

Blood samples were collected immediately before ECLS initiation and again 24 h later. IL6 levels were analyzed using electrochemiluminescence (Cobas e411, Roche Diagnostics, Rotkreuz, Switzerland). Serum levels of IL-6 were measured daily in fresh samples using a standardized protocol in the hospital’s central laboratory without batch analysis. The lower limit of detection (LoD) for IL-6 was 1.5 pg/mL, and the coefficient of variation (CV) ranged from 2% to 3%, depending on the concentration.

In contrast, IL-8, TNF-α, and soluble interleukin-2 receptor (sIL2R) were measured from frozen serum samples and analyzed in weekly batches. IL-8 and TNF-α were quantified using chemiluminescence-based immunoassays on the Immulite 1000 analyzer (Siemens Healthcare Diagnostics, Erlangen, Germany), with a LoD of 2 pg/mL and inter-assay CV of 2.8–9.5% for IL-8, and a LoD of 1.7 pg/mL with a CV of 2.7–7.8% for TNF-α. sIL2R was measured using a two-site chemiluminescent immunometric assay on the same platform, with a LoD of 5 U/mL and a CV ranging from 3.9% to 21%, depending on the concentration.

### Statistical methods

The collected data were analyzed using IBM SPSS 29 (SPSS, Inc., Chicago, IL, USA) and SigmaPlot 14.0 (Systat Software Gmbh, Erkrath, Germany). A *p*-value of ≤0.05 was considered statistically significant in all analyses.

To analyze differences between groups of patients Mann–Whitney *U* test for nonparametric data was chosen and a chi-square test for categorical variable were performed. To evaluate a prognostic impact on patient survival, survival analysis by Log-Rank was provided. Odds ratios were calculated during analysis of risk factors and their strength and were derived from 2 × 2 contingency tables. To determine the correlation of non-parametric data, we calculated Spearman's rank correlation coefficient (*r*_s_). To interpret the strength of Spearman’s rank correlation, we used Cohen’s (1988) guidelines. According to Cohen (1988), the interpretation of the correlation coefficient *ρ* is as follows: small (weak) correlation: |*ρ*| = 0.10, medium (moderate) correlation: |*ρ*| = 0.30, large (strong) correlation: |*ρ*| = 0.50.

## Results

The present study included 546 patients who underwent extracorporeal life support at a tertiary care center in the period from 2013 until 2023. The median follow-up was 9 (IQR 2; 434) days. The duration of ECLS was 3 (IQR 2; 5.25) days in median. 317 (58.06%) patients could be weaned from ECLS, 30-day survival and 1 year survival was 44.0% (217 patients) and 31.4% (144 patients) respectively.

Coronary artery disease was the most frequent underlying diagnosis leading to ECLS (54.8% of cases). Apart from dilated cardiomyopathy, pulmonary embolism, rhythmic events (refractory ventricular fibrillation and ventricular tachycardia), and sepsis, a variety of miscellaneous conditions (hemorrhagic shock, intoxication, near drowning, hypothermia and anaphylactic shock) complete the underlying diagnoses.

Patients were divided into two groups: the first group consisting of 358 patients, who received ECLS in-hospital as part of eCPR. The second group consisted of 188 patients, who received ECLS as part of eCPR in an out-of-hospital setting.

**Fig. 1:**
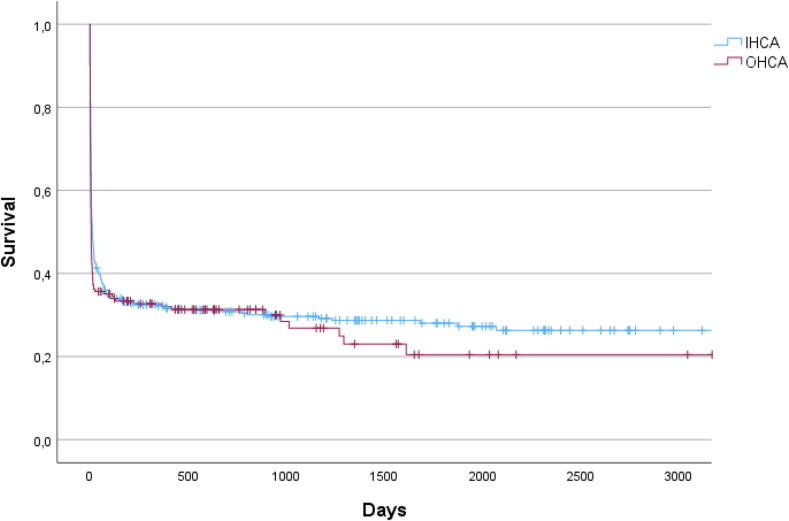
Kaplan-Meier survival curves between in-hospital and out-of-hospital groups (IHCA – in hospital cardiac arrest, OHCA – out of hospital cardiac arrest).

There was no statistical difference concerning 30 day (*p* = 0.081) and 1 year survival (*p* = 0.219) between the two groups (Fig. 1).

### Cytokines

**Fig. 2:**
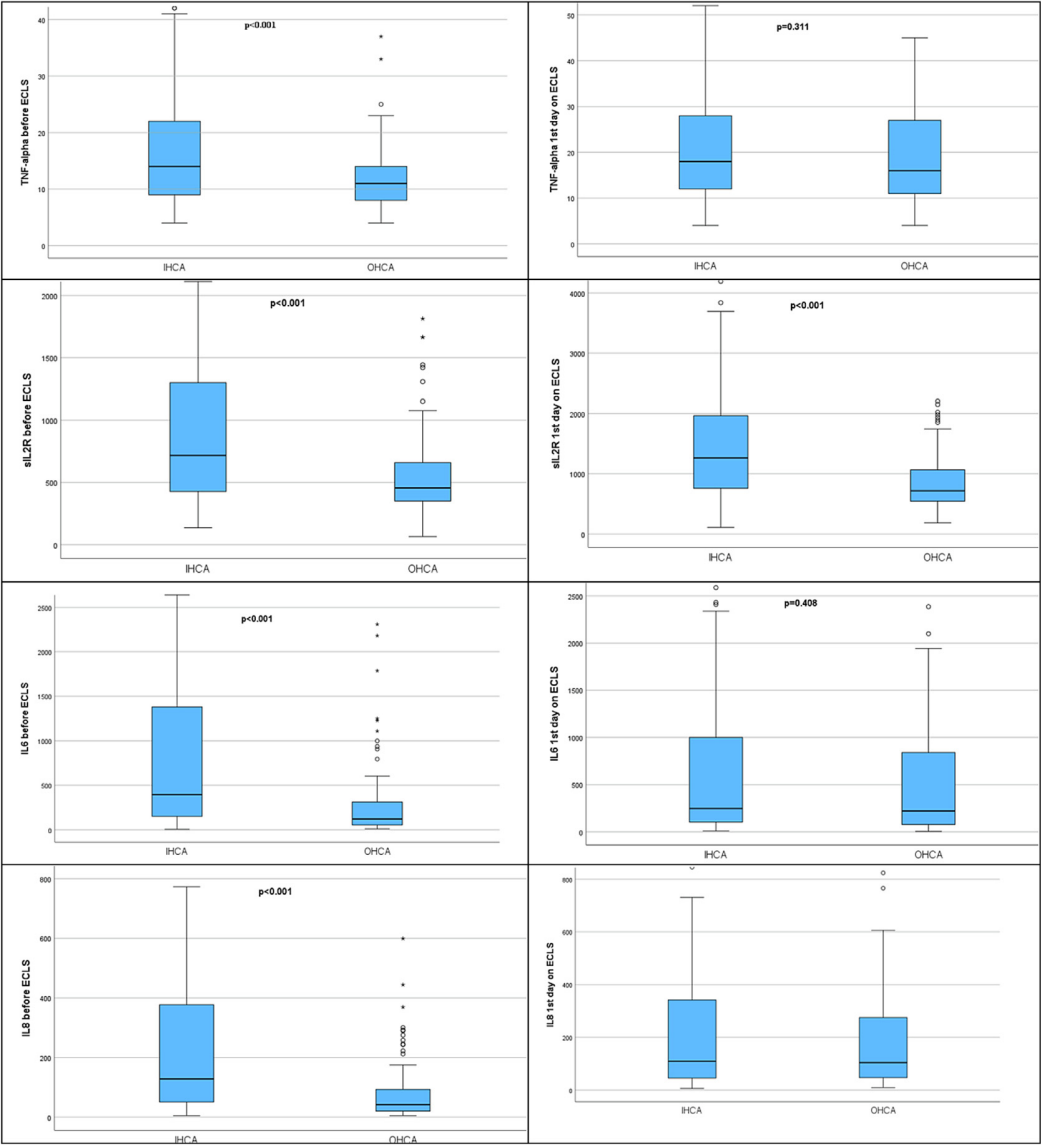
Cytokines in the relationship between patients who received in-hospital and out-of-hospital ECLS (IHCA – in hospital cardiac arrest, OHCA – out of hospital cardiac arrest, TNF-alpha – tumor necrosis factor alpha, sIL2R – soluble interleukin 2 receptor, IL6 – interleukin 6, IL8 – interleukin 8).

The serum levels of IL6, IL8, TNF-alpha, and sIL2R prior to ECLS implantation was significantly higher in the group of patients undergoing mechanical circulatory support in the hospital. On the first day after implantation of ECLS only sIL2R levels were still significantly elevated in both groups (Table 3, Fig. 2). There were no significant differences in the levels of TNF-alpha, IL6 and IL8 on the first day of ECLS between the two groups of patients. Analyzing the dynamics of cytokines, it turned out that IL6 and IL8 showed a significant increase during the first day on ECLS in the OHCA patients whereas significant reduction in the level of these two cytokines revealed in the IHCA patients. TNF-alpha and sIL2R showed a significant increase in both groups during the first 24 h after eCPR. (Table 3).

Analyzing the correlation between resuscitation time before eCPR initiation and cytokine levels directly before ECLS implantation for OHCA patients a strong correlation with the CPR time for IL6 and IL8 (*r*_s_ = 0.495, *p* < 0.001 and *r*_s_ = 0.45, *p* < 0.001, respectively) and a moderate correlation with sIL2R before and after 24 h (*r*_s_ = 0.376, *p* < 0.001 and *r*_s_ = 0.295, *p* < 0.001 respectively) could be shown. Analyzing the in-hospital group, correlation between CPR duration and IL6 prior to (*r*_s_ = 0.222, *p* = 0.002) and 24 h (*r*_s_ = 0.245, *p* = 0.001) after ECLS initialization was demonstrated. Furthermore, IL8 serum levels correlated significantly with CPR duration after 24 h of ECLS therapy in both groups (Table A1).

### Neurological outcomes

Neurological status was assessed in a subgroup of 376 patients, in which the clinical course allowed neurological assessment. In the IHCA group, there were a significantly better cerebral performance (CPC 1 and CPC 2 in 136 IHCA patients (60.0%) vs. 60 OHCA patients (40.3%), *p* < 0.001) and a lower probability of cerebral death (odds ratio 0.516; 95% CI 0.339–0.785, *p* = 0.003) compared to patients who received ECLS in an OHCA setting.

Analyzing the correlation of cytokine levels with deterioration of cerebral performance, in the group of patients who received ECLS outside the hospital, there was a significant correlation between IL6, IL8 and TNF-alpha. Also the strength of the association between cytokine levels and CPC scores increased over the first 24 h of ECMO, with IL-6, IL-8, and TNF-α showing a marked correlation with neurological outcomes at 24 h (Table A3). The IHCA group demonstrated a significant correlation with the increase in severity of cerebral dysfunction by IL6 and IL8 and also showed an increase in correlation strength 24 h after eCPR initiation (Table A3).

### Organ failure

With cardiocirculatory failure leading to ECLS therapy, all patients were defined to present with at least 1 failing organ system. In 156 (28.6%) patients, no further organ dysfunction occurred, including 68 (19.0%) IHCA patients versus 88 (46.8%) OHCA patients (*p* < 0.001). Two organ dysfunction was diagnosed in 232 (42.5%) patients, 152 (42.5%) IHCA patients vs 80 (42.5%) OHCA patients (*p* < 0.983). Dysfunction affecting three organs was diagnosed in 117 (21.4%) patients, 99 IHCA (27.7%) vs 18 OHCA (9.6%) patients respectively (*p* < 0.001). Four organ systems were affected in 41 (7.5%) patients, including 39 (10.9%) IHCA patients and 2 (1.1%) OHCA patients (*p* < 0.001).

In the group of patients who received ECLS in the hospital, MODS was significantly more frequent compared to the group who received ECMO out-of-hospital, 138 (38.5%) vs 20 (10.6%) patients (*p* < 0.001).

Analyzing the risk for the occurrence of specific organ dysfunctions the IHCA group presented with an increased risk for developing acute hepatic dysfunction (odds ratio 4.193; 95% CI 2.166–8.117, *p* < 0.001), acute renal failure (odds ratio 4.168; 95% CI 2.65–6.557, *p* < 0.001) and acute respiratory distress syndrome (odds ratio 2.639; 95% CI 1.836–3.795, *p* < 0.001) when compared to OHCA patients (Table 1).

**Table 1:** Baseline characteristics and outcome data.

Category	All Patients			*p*-value
		In-hospital	Out of hospital	
Number of Patients	546	358	188	
Age, years	58.4 [49.9; 66.73]	59.45 [50.52; 67.55]	57.3 [49.55; 65.75]	0.084
Women n, (%)	142 (26.0%)	104 (29.1%)	38 (20.2%)	**0.025**
BMI kg/cm2	26.9 [24.32; 30.07]	27.31 [24.44; 30.28]	26.39[24.22; 29.41]	0.552
CPR time to ECLS initiation, min	42 [30; 64]	40 [23.5; 65.5]	45 [34.25; 62]	**0.009**
ECLS weaned	317 (58.06%)	208 (58.1%)	109 (58%)	0.978
Follow-up, days	9 [2; 434]	12 [2; 526.25]	6 [2; 367]	0.055
Days of ECLS	3 [2; 5.25]	4 [2; 6]	3 [2; 4]	**<0.001**
30 days survival	217 (44.0%)	150 (46.3%)	67 (39.6%)	0.081
1st year survival	144 (31.4%)	97 (31.8%)	47 (30.5%)	0.219

*Neurological outcome of 376 Patients*				
CPC 1–2	196 (52.1%)	136 (60.0%)	60 (40.3%)	**<0.001**
CPC 3–4	15 (4.0%)	6 (2.6%)	9 (6.0%)	**<0.001**
CPC 5	165 (43.9%)	85 (37.4%)	80 (53.7%)	**<0.001**

*Organ failure*				
Multi-organ failure*	158 (28.9%)	138 (38.5%)	20 (10.6%)	**<0.001**
ALD	85 (15.6.7%)	74 (20.6%)	11 (5.9%)	**<0.001**
AKF	179 (32.8%)	151 (42.2%)	28 (14.9%)	**<0.001**
ARDS	325 (59.5%)	242 (67.6%)	83 (44.1%)	**<0.001**

*Underlying disease*				
CAD *n*, (%)	299 (54.8%)	193 (53.9%)	106 (56.4%)	0.581
DCM *n*, (%)	17 (3.1%)	12 (3.6%)	5 (2.7%)	0.658
PE *n*, (%)	67 (12.3%)	50 (14.0%)	17 (9.0%)	0.096
VT/VF *n*, (%)	93 (17.0%)	48 (12.6%)	45 (23.9 %)	**0.002**
Septic shock *n*, (%)	12 (2.2%)	11 (3.1%)	1 (0.5%)	0.054**
Other *n*, (%)	58 (10.6%)	44 (12.3%)	14 (7.4%)	0.081

Data are expressed as median and interquartile range [25th–75th] or number (percentage).

BMI – Body-Mass-Index, CPR – cardiopulmonary resuscitation, ECLS – extracorporeal life support, CAD – coronary artery disease, DCM – dilated cardiomyopathy, PE – pulmonary embolism, CPC – cerebral performance category, ALD – acute liver dysfunction, AKF – acute kidney failure, ARDS – acute respiratory distress syndrome, VT/VF – refractory ventricular fibrillation and ventricular tachycardia.

* Multi-organ failure was defined as impairment of three or more organ systems.

** Taking into account that in some groups the frequency of the value was less than 5, the test cannot be considered as reliable.

### Hemodynamics

Comparing the initial hemodynamic situation MAP was significantly higher in patients undergoing ECLS implantation in the hospital compared to the group undergoing ECMO initiation outside the hospital (55 mmHg vs 35 mmHg, *p* < 0.001).

On the first day after ECLS implantation, the hemodynamic status was stabilized without a significant difference in MAP in both groups (63 mmHg vs 63 mmHg, *p* = 0.944). However, the requirement of catecholamines based on VIS was significantly higher in the group of patients who were hospitalized prior to eCPR (9.259 vs 4.167, *p* < 0.001, Table 2).

**Table 2:** Hemodynamic parameters before and during ECLS.

Category	All Patients	In-hospital	Out-of-Hospital	*p*-value
Number of Patients	546	358	188	
MAP before ECLS, mmHg	40 [34; 55]	48 [35; 61]	35 [30; 40]	**<0.001**
MAP at 1st day after ECLS mmHg	64 [59;70]	64 [59; 70]	64 [59; 71]	0.835
Lactate before ECLS, mg/dl	102 [65; 137]	99 [59; 138]	106 [73; 137]	0.085
Lactate at 1st day after ECLS. mg/dl	38.5 [20.75; 75]	38 [21;75]	39 [20; 75]	0.964
Vasoactive-inotropic score before ECLS	33.3 [0; 81.6]*	60.1 [27.8; 109.8]	−*	
Vasoactive-inotropic score after ECLS	5.064 [0; 18.265]	9.259 [2.097; 22.862]	4.167 [0.00; 10.296]	**<0.001**

Data are expressed as median and interquartile range [25th–75th].

MAP – mean arterial pressure, BMI – Body-Mass-Index, ECLS – extracorporeal life support.

* There are no all data on the amount of catecholamines in this group.

**Table 3:** Analysis of interleukins between in-hospital and out-of-hospital patients.

	In-hospital	Out-of-hospital	*p*-value *IHCA* vs *OHCA*
Preimplantation TNF-alpha, pg/ml	14 [9; 23]	11 [8; 14]	***<0.001***
1st day TNF-alpha, pg/ml	17 [12; 27]	16 [11; 27]	*0.311*
*p*-value before vs after ECLS	***<0.001***	***<0.001***	

Preimplantation sIL-2R, U/ml	707 [423; 1274]	456.5 [346.0; 686.75]	***<0.001***
1st day sIL-2R, U/ml	1140.5 [749.5; 1927]	727 [546; 1082]	***<0.001***
*p*-value before vs after ECLS	***<0.001***	***0.001***	

Preimplantation IL6, pg/ml	420 [144.5; 1410]	136.5 [59; 320.25]	***<0.001***
1st day IL6, pg/ml	243 [104; 1024]	231 [77.5; 995]	*0.408*
*p*-value before vs after ECLS	***0.01***	***<0.001***	

Preimplantation IL8, pg/ml	124 [48.5; 393]	42 [21.25; 88]	***<0.001***
1st day IL8, pg/ml	111.5 [45; 330.75]	111 [47; 310]	*0.903*
*p*-value before vs after ECLS	***0.008***	***<0.001***	

Data are expressed as median and interquartile range [25th–75th].

TNF-alpha – tumor necrosis factor alpha, sIL2R – soluble interleukin-2 receptor, IL6 – interleukin-6, IL8 – interleukin-8.

Correlating cytokine levels with VIS on the first day after ECLS initiation, a weak correlation was found with sIL2R (*r*_s_ = 0.225, *p* < 0.001) in IHCA patients and a moderate correlation with sIL2R and TNF-alpha (*r*_s_ = 0.292, *p* < 0.001 and *r*_s_ = 0.264, *p* = 0.001) in OHCA patients. Interleukin 8 demonstrated a moderate correlation in both groups. The most distinct correlation was shown by IL6, which showed a pronounced relationship with VIS, especially in the IHCA patients (*r*_s_ = 0.421, *p* < 0.001. Table A2).

Lactate levels before and after ECLS initiation didn’t differ between the two groups of patients. However, lactate levels dropped significantly after ECLS implantation in both groups (Table 2).

## Discussion

Despite previous studies comparing patients undergoing eCPR during IHCA vs OHCA and factors influencing the outcome,^3^, ^9^, ^8^, ^18^ as well as research on cytokine levels and their impact on neurological outcome in patients with eCPR in an out-of-hospital setting,^19^ the evidence base for the use of eCPR and the understanding of pathophysiological processes remains rather scarce. Hitherto, there have been no studies comparing both groups of patients regarding cytokines and their impact on the clinical course. In addition, studying the dynamics of the inflammatory status and clinical course of patients with acute ischemia compared to patients with a higher likelihood of chronic tissue ischemia is of interest.

We compared two groups of patients, the first group, who had already been in the hospital for a certain condition (i.e. severe pre-existing illnesses) and, most likely due to progressive or rapid deterioration, underwent CPR with the need of ECLS. The second group of patients who, due to a predominantly sudden deterioration of their underlying disease, were resuscitated outside the hospital and underwent extracorporeal circulatory support due to the inability to achieve ROSC. After the initiation of ECLS, all patients were sufficiently perfused and the development of PRS was analyzed. Specifically brain damage, hemodynamic disorders, and other organ dysfunctions, including the development of multiple organ failure in both groups of patients was examined through the prism of cytokines.

The IHCA group of patients had a more pronounced proinflammatory status before ECLS implantation, probably due to the underlying disease, various types of interventions and procedures, as well as chronic systemic tissue ischemia in a longstanding underlying condition. In the OHCA group of patients, the initial proinflammatory response was significantly lower.

Following ECLS initialization the out-of-hospital group showed a significant increase of IL6, IL8, TNF-alpha and sIL2R, probably caused by acute ischemia due to no-flow and low-flow time during resuscitation, ensuing reperfusion injury as well as other presumed factors including ECLS initiation, interventional diagnostic and therapeutic procedures. In contrast, in the in-hospital group of patients, a significant decrease of IL6 and IL8 was noted as a possible result of sufficient perfusion of organs and organ systems and, consequently, a decrease in the inflammatory status.

Patients who received ECLS out-of-hospital, due to an acute event, were initially more difficult to stabilize hemodynamically, as indicated by the significantly lower MAP in this group. However, after 24 h, both groups were stabilized with respect to hemodynamics, and the out-of-hospital group presented with significantly lower VIS to maintain this stabilization. This may be explained by the fact that OHCA patients had a significantly lower baseline level of proinflammatory activation. The correlation between VIS after 24 h and cytokine levels before and after initiation of ECLS points this out.

According to this observation a study of patients undergoing CPR without ECLS also demonstrated an increase in catecholamines demand accompanied by an elevation of IL8 and TNF-alpha values.^20^ Moreover, IL6 in vivo studies have demonstrated arteriolar dilation, which leads to systemic vasoplegia,^21^ which may be the explanation behind the increase in vasoconstrictors.

The risks of acute respiratory distress syndrome, acute hepatic dysfunction, and acute renal failure turned out to be significantly higher in the in-hospital group. Consequently, multiple organ failure was also significantly more likely in patients who underwent in-hospital ECMO implantation. One of the factors that could contribute to this is a raised proinflammatory level of cytokines at the time of ECLS initiation that facilitated the development of multiple organ dysfunction, a interdependency, which has already been described.^14^, ^13^

A favourable neurological outcome of IHCA vs OHCA patients, as well as a lower risk of brain death, has already been noted in previous eCPR studies.^9^ Factors contributing to this are the shorter duration of CPR prior to ECLS initiation, as well as a higher skill level and a bigger range of specialists in the hospital performing CPR during low-flow and mostly absent or minimal no-flow time. Concerning inflammation and neurological outcome IL6, IL8, and TNF-alpha were strongly correlated 24 h after ECLS initialization with the neurological status of out-of-hospital patients, which is likely a reflection of the impact of acute ischemia on the central nervous system. In the group of patients who underwent mechanical circulatory support in the hospital, the correlation of neurological damage with IL6 was less pronounced, in comparison a stronger correlation with IL8 was observed, which is likely to be reflection of the more pronounced association to neurological impairments in the setting of prevalent pro-inflammatory activation in our study. Similarpcas association of IL6 and IL8 have already been shown in patients presenting with ROSC following CPR with and without ECLS.^20^, ^18^

Also, the duration of CPR prior to ECLS initialization correlated with cytokine levels. In patients resuscitated outside the hospital, there was a strong correlation between TNF-alpha, IL6 and IL8 with the duration of low-flow time and a decrease in correlation strength 24 h after stable sufficient perfusion was established.

One of the factors that could also affect the level of cytokines between the groups of patients is the significantly different proportion of patients with VF/VT. In the OHCA group, it was larger, which could also affect both the level of cytokines and their consequences, due to different management of patients (both medication and electrophysiological) during resuscitation.

Although the cytokines, we studied, are not the sole triggers for the components of PRS, they appear to play a possible role in its development. The considerable association of cytokines on the clinical course and outcomes of patients may be subject to further discussion about whether their role is purely diagnostic and prognostic or whether they can serve as a valuable therapeutic target. Former research endeavors aimed at decreasing serum interleukin levels for therapeutic goals have generated outcomes that remain a matter of debate.^22^, ^23^, ^24^, ^25^

### Limitations

Possible limitations of this study are the period of 10 years during which data were collected, taking into account changing features of ECLS patient management and technical tools over time. Furthermore, the underlying patient population presents rather heterogenous. The retrospective nature of the analysis as well as reporting the experience of a single center may also be a restriction for definitive conclusions. As an observational study, it identifies associations but cannot confirm causality. Findings are hypothesis-generating and require validation in future prospective studies. Due to multiple testing, a risk of type I errors cannot be excluded.

## Conclusion

IL-6, IL-8, and TNF-alpha are closely associated with key components of the post-resuscitation syndrome (PRS) and increase with the duration of resuscitation. In patients without a preexisting proinflammatory burden, these cytokines show a stronger correlation with PRS features. Furthermore, in-hospital initiation of eCPR is followed by an initial decline in proinflammatory cytokine levels during the subsequent clinical course.

## Declaration of AI and AI-assisted technologies in the writing process

During the preparation of this work the author used DeepL Write in order to improve readability and language. After using this tool, the authors reviewed and edited the content as needed and takes full responsibility for the content of the publication.

## Data availability statement

The datasets used and/or analyzed during the current study are available from the corresponding author on reasonable request.

## CRediT authorship contribution statement

**Kostiantyn Kozakov:** Writing – review & editing, Writing – original draft, Visualization, Methodology, Investigation, Formal analysis, Data curation, Conceptualization. **Zdenek Provaznik:** Writing – review & editing, Supervision, Resources, Investigation, Formal analysis. **Maik Foltan:** Writing – review & editing, Resources, Data curation. **Jing Li:** Writing – review & editing. **Walter Petermichl:** Writing – review & editing, Resources. **Christian Stadlbauer:** Writing – review & editing. **Sigrid Wiesner:** Writing – review & editing. **Dzmitry Turtsevich:** Writing – review & editing, Resources. **Andreas Keyser:** Writing – review & editing, Supervision. **Leopold Rupprecht:** Writing – review & editing, Supervision, Resources. **Christof Schmid:** Writing – review & editing, Validation, Supervision, Resources, Investigation. **Simon Schopka:** Writing – review & editing, Validation, Supervision, Resources, Project administration, Investigation, Formal analysis, Data curation, Conceptualization.

## Ethics approval

The study was conducted according to the principles of the Declaration of Helsinki and was approved by the Ethics Committee of the University Medical Center of Regensburg (Reference number: 23-3595-104; 12. December 2023).

## Declaration of competing interest

The authors declare that they have no known competing financial interests or personal relationships that could have appeared to influence the work reported in this paper.

## Figures and Tables

**Table A1:** Correlation between CPR time to initiation of ECLS and cytokines value.

	CPR time to ECLS initiation, min		
	In-hospital	Out-of-hospital	
Preimplantation TNF-alpha	0.0109	**0.376**	Correlation coefficient
	0.886	**<0.001**	*p*-value
1st day TNF-alpha	0.107	**0.295**	Correlation coefficient
	0.172	**<0.001**	*p*-value

Preimplantation sIL-2R	−0.178	0.142	Correlation coefficient
	0.018	0.0608	*p*-value
1st day sIL-2R	−0.0813	0.136	Correlation coefficient
	0.302	0.097	*p*-value

Preimplantation IL6	**0.222**	**0.492**	Correlation coefficient
	**0.002**	**<0.001**	*p*-value
1st day IL6	**0.245**	**0.24**	Correlation coefficient
	**0.001**	**0.003**	*p*-value

Preimplantation IL8	0.107	**0.45**	Correlation coefficient
	0.155	**<0.001**	*p*-value
1st day IL8	**0.214**	**0.252**	Correlation coefficient
	**0.006**	**0.002**	*p*-value

CPR – cardiopulmonary resuscitation, TNF-alpha – tumor necrosis factor alpha, sIL2R – soluble interleukin-2 receptor, IL6 – interleukin-6, IL8 – interleukin-8, ECLS – extracorporeal life support.

**Table A2:** Correlation between cytokines and vasoactive-inotropic score on 1st day of ECLS.

	VIS on 1st day		
	In-hospital	Out-of-hospital	
Preimplantation TNF-alpha	0.100	**0.204**	Correlation coefficient
	0.105	**0.01**	*p*-value
1st day TNF-alpha	**0.136**	**0.264**	Correlation coefficient
	**0.027**	**0.001**	*p*-value

Preimplantation sIL2R	**0.143**	**0.219**	Correlation coefficient
	**0.02**	**0.006**	*p*-value
1st day sIL2R	**0.225**	**0.292**	Correlation coefficient
	**<0.001**	**<0.001**	*p*-value

Preimplantation IL6	**0.196**	**0.184**	Correlation coefficient
	**<0.001**	**0.02**	*p*-value
1st day IL6	**0.421**	**0.329**	Correlation coefficient
	**<0.001**	**<0.001**	*p*-value

Preimplantation IL8	**0.178**	**0.216**	Correlation coefficient
	**0.004**	**0.007**	*p*-value
1st day IL8	**0.288**	**0.346**	Correlation coefficient
	**<0.001**	**<0.001**	*p*-value

TNF-alpha – tumor necrosis factor alpha, sIL2R – soluble interleukin-2 receptor, IL6 – interleukin-6, IL8 – interleukin-8, VIS – vasoactive-inotropic score, ECLS – extracorporeal life support.

**Table A3:** Correlation between cytokines and cerebral performance category score.

	CPC score		
	In-hospital	Out-of-hospital	
Preimplantation TNF-alpha	0.091	**0.344**	Correlation coefficient
	0.217	**<0.001**	*p*-value
1st day TNF-alpha	**0.159**	**0.417**	Correlation coefficient
	**0.034**	**<0.001**	*p*-value

Preimplantation sIL-2R	**−0.164**	0.081	Correlation coefficient
	**0.027**	0.342	*p*-value
1st day sIL-2R	−0.062	0.133	Correlation coefficient
	0.407	0.132	*p*-value

Preimplantation IL6	**0.195**	**0.329**	Correlation coefficient
	**0.007**	**<0.001**	*p*-value
1st day IL6	**0.289**	**0.409**	Correlation coefficient
	**<0.001**	**<0.001**	*p*-value

Preimplantation IL8	**0.162**	**0.188**	Correlation coefficient
	**0.029**	**0.027**	*p*-value
1st day IL8	**0.356**	**0.533**	Correlation coefficient
	**<0.001**	**<0.001**	*p*-value

TNF-alpha – tumor necrosis factor alpha, sIL2R – soluble interleukin-2 receptor, IL6 – interleukin-6, IL8 – interleukin-8, CPC – cerebral performance category.

## Appendix A

See Table A1, Table A2, Table A3.
